# Context Memory Discrimination and Recognition in Humans: Development of a Novel Cognitive Task

**DOI:** 10.2196/87362

**Published:** 2026-06-24

**Authors:** Sonalee Arun Joshi, Rachel John, Ivy Fei Tso, Carly A Lasagna, Amanda Hicks, Craig Stark, Shauna Stark, Anthony King, James Abelson, Elizabeth R Duval, Israel Liberzon

**Affiliations:** 1 VISN 4 MIRECC, Cpl. Michael J Crescenz VA Medical Center, Philadelphia, PA, United States; 2 Department of Psychiatry, University of Michigan, 4250 Plymouth Road, Ann Arbor, MI, 48109, United States, 1 734 764 0231; 3 Department of Psychiatry and Behavioral Health, The Ohio State University, Columbus, OH, United States; 4 Thriving Minds Behavioral Health, Chelsea, MI, United States; 5 Neurobiology and Behavior School of Biological Science, University of California, Irvine, Irvine, CA, United States; 6 Department of Psychiatry, Texas A&M University, Bryan, TX, United States

**Keywords:** context, memory, discrimination, recognition, task development

## Abstract

**Background:**

When confronted with ambiguous stimuli, the ability to utilize contextual information is crucial for survival. Context processing involves the ability to discriminate new information from previously encountered information and to recognize something as previously encountered, even briefly or partially. Deficits in context processing are a key feature across a number of psychiatric conditions. Existing tasks only examine the discrimination and recognition of cues as opposed to contextual information. Thus, new tasks using complex scenes are urgently needed.

**Objective:**

We developed the Context Discrimination and Recognition Task (CDRT) and established baseline performance in healthy adults.

**Methods:**

Final analyses included 44 participants (mean age 30.27, SD 11.78) who completed the CDRT to characterize memory performance for (1) discrimination between previously viewed and never viewed complex scenes (Discrete Discrimination Score [DDS]), (2) recognition of previously viewed complex scenes (Discrete Recognition Score [DRS]), (3) sensitivity to distinctions across a gradient of complex scenes (width), and (4) bias in recognition of complex scenes (bias). Pearson correlations were conducted to examine associations between these scores.

**Results:**

The results revealed a significant positive relationship between DDS and DRS scores, *r*(41)=0.71, *P*<.001, and between DDS and modified recognition scores, *r*(41)=0.71, *P*<.001. Width was also negatively correlated with DDS, *r*(41)=−0.31, *P*=.04; DRS, *r*(41)=−0.47, *P*=.001; and DRSmid, *r*(41)=−0.35, *P*=.02

**Conclusions:**

We found that the CDRT is sensitive to a range of discrimination and recognition levels and is uniquely positioned to probe context processing. Additionally, both recognition and discrimination scores increased with decreased ambiguity of complex scenes, demonstrating the sensitivity of the task in detecting variability of performance across participants. Better recognition of complex scenes was associated with greater sensitivity to differences between contexts. This novel task can be used to assess memory-associated processes for complex scenes in future studies aiming to elucidate neural functions underlying context processing in psychiatric conditions.

## Introduction

The ability to properly use information about environmental context to modulate emotional and behavioral responses is crucial to survival (eg, a lion is dangerous in the wild but safe in a zoo). Deficits in the utilization of contextual information have been implicated in the development and maintenance of a number of psychiatric conditions, pointing toward specific neural circuits and neurobiological processes involved. People with anxiety exhibit heightened acquisition of fear in contexts where a threat was encountered and are more prone to generalize this fear response to nonthreatening contexts [1-3]. Patients with posttraumatic stress disorder (PTSD) have difficulty recognizing when ambiguous cues might be safe or dangerous based on the context in which the cue is presented [4] (eg, reacting to the sound of fireworks during a celebration as if they were explosions in a warzone). Substance use disorders have been linked to increased reactivity to substance-related cues in social versus neutral contexts, as well as an increased risk of overdose in a new context [5-8]. Context processing deficits in aging populations are accelerated by neurodegenerative conditions (eg, mild cognitive impairment, Alzheimer disease, and Parkinson disease) and further reduce the ability to use environmental information to modulate behavior [9]. Thus, continuous recognition tasks such as the Memtrax Task have been developed to examine general episodic memory performance associated with aging [10].

Detecting contextual deficits can help with the identification of specific neural circuits involved in a number of psychopathologies, but very few paradigms to date directly assess context processing. The goal of this study was to develop a novel task designed to probe context-based discrimination and recognition abilities using complex scenes and to quantify performance in a sample of healthy adults. Establishing baseline performance in a nonclinical sample is a critical first step for subsequent research aimed at quantifying context processing associated with psychopathology.

Effective context discrimination and recognition require well-balanced hippocampal-dependent processes of pattern separation (PS) and pattern completion (PC) [11,12]. PS is measured by one’s ability to *discriminate* partially similar but overall different patterns and *encode* them as separate memory representations [13-15]. PC is the ability to *identify* a correct pattern when only partial information is available [16]. Imbalances in context-based PS and PC may contribute to the “misidentification” of context as erroneously “dangerous,” leading to behavioral consequences. Previous research has linked deficits in PS and PC to several psychiatric conditions, including anxiety [13] and PTSD [11,17,18]. For example, insufficient PS and excessive PC contribute to threat overgeneralization [11], leading to hypervigilance, hyperarousal, and fear responses in realistically safe situations, as well as excessive risk-taking in dangerous situations [4,19,20].

Tasks such as the Mnemonic Similarity Task (MST) [15,21,22] have been developed to probe PS and PC using simple objects; however, tasks utilizing complex scenes are needed for greater ecological validity. Complex scenes more closely resemble situations in the real world that require one to simultaneously and rapidly encode or recall multiple sources of contextual information based on prior memory to inform behavioral responses. Tasks using simple items have successfully captured discrimination as a proxy for PS; however, they are not well-suited to quantify the PC-like process of recognizing a stimulus as “old” based on *partial*, previously seen features. The proper identification of danger or safety requires a complex balance between taking quick action after recognizing a tail in the grass as part of a lion and the ability to override the PC-based threat response by PS—recognizing that the tail might resemble a lion’s, but it is too small and might belong to the neighbor’s poodle. Examining responses to a gradient of complex scenes in a task that simultaneously assesses both PS and PC could be more sensitive to deficits in the use of contextual information associated with psychopathology [1-3,5,6,10,23].

In this study, we describe the development of the Context Discrimination and Recognition Task (CDRT), designed specifically to probe context-based PS and PC using a continuum of complex scenes. We detail discrete metrics to examine both discrimination as a proxy for PS and recognition as a proxy for PC. Additionally, we outline continuous metrics used to explore nuances in discrimination and recognition across a continuum of complex scenes. Consistent with the development of previous tasks probing PS and PC (eg, MST; [24]), this study aimed to characterize performance on this task in a healthy adult sample to establish a baseline for future studies examining differences associated with various clinical conditions.

## Methods

### Participants

Data from 44 participants, ranging in age from 18 to 66 years (mean 30.27, SD 11.78), were included in this study (see Table 1 for sample demographics). Eligibility was determined by a telephone screen and a brief clinical interview via the Mini-International Neuropsychiatric Interview [25] performed by a licensed psychologist or a master’s-level clinician supervised by the psychologist. Inclusion criteria included no current or past diagnosis of significant medical, neurological, or psychiatric conditions; no use of psychotropic medications; and ability and willingness to speak English and provide informed consent. Participants were recruited from the University of Michigan campus and the larger Ann Arbor, MI, community using flyers and online advertisements.

**Table 1.** Demographics and descriptive statistics.

Characteristic	All (N=44)	Men (n=12)	Women (n=32)
Age (y), mean (SD)	29.9 (11.9)	36.5 (16.7)	27.4 (8.6)
Race/ethnicity, n (%)			
White/Caucasian	31 (71)	9 (75)	22 (69)
Black/African American	2 (5)	0 (0)	2 (6)
Asian	6 (14)	2 (17)	4 (13)
Latinx/Hispanic	2 (4)	0 (0)	2 (6)
Indigenous/Native American	2 (4)	0 (0)	2 (6)
Not specified	1 (2)	1 (8)	0 (0)
Education			
Associate degree	3 (7)	0 (0)	3 (9)
Some college	18 (41)	2 (17)	7 (22)
Bachelor’s degree	18 (41)	7 (58)	11 (34)
Some graduate education	5 (11)	0 (0)	16 (5)
Master’s degree	7 (16)	3 (25)	13 (4)
Doctoral degree	1 (2)	0 (0)	3 (1)
Not specified	1 (2)	0 (0)	1 (31)

### Ethical Considerations

All procedures were approved by the institutional review board at the University of Michigan and the VA Ann Arbor Healthcare System (2016‐020133 approved by VA Ann Arbor Healthcare System Human Studies Committee; HUM00107882 approved by the University of Michigan IRB-MED). All participants included in this study provided informed consent and received monetary compensation for their participation. We attest to maintaining the privacy and confidentiality of participants’ data and identities. This study was carried out in accordance with the provisions of the World Medical Association Declaration of Helsinki.

### Procedures

#### Assessment

No participants met *Diagnostic and Statistical Manual of Mental Disorders, Fifth Edition* (*DSM-5*) diagnostic criteria for current or past psychiatric conditions. Demographic information collected included age, self-identified gender, race, ethnicity, and highest level of education achieved. Education was grouped categorically and included as a covariate in analyses. A subset of participants also completed additional cognitive tasks and underwent magnetic resonance imaging scanning as a pilot component of data collection (not reported in the present report).

#### Task Development

Building on previous research examining discrimination and recognition of simple items, the CDRT was developed to specifically examine discrimination and recognition of complex scenes mirroring real-world contextual settings.

##### Target Images

**Figure 1.**
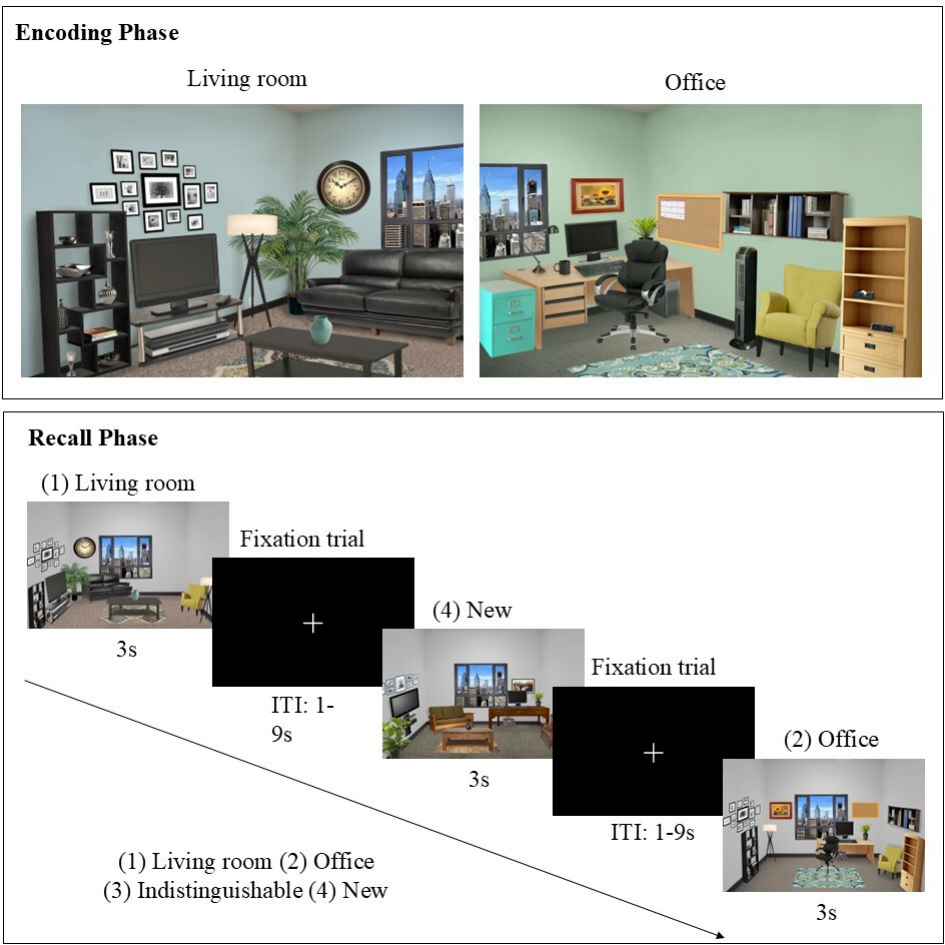
Encoding and recall phases of the Context Discrimination and Recognition Task (CDRT). (A) Target images of the living room and office were presented during the encoding phase. Participants passively viewed each target image twice in a random order for 5 seconds each. (B) Sample trials from the recall phase of the CDRT. Participants completed 3 runs of the recall by indicating whether each image was most similar to the living room or office target images, indistinguishable (equally similar to both target images), or a new image. Each of the 12 similar images was presented 4 times per run, resulting in 48 trials per run for a total of 144 trials during the recall phase.

Two target images were created to depict a room containing 10 items (ie, furniture) that either represented a prototypical “living room” or a prototypical “office” (Figure 1A). The target images were oriented at opposite angles and included the same window and view of a skyline to suggest that the viewer could be looking at 2 different rooms in the same building in order to minimize cues external to the rooms that could influence perception and memory.

##### Living Room-Office Continuum

**Figure 2.**
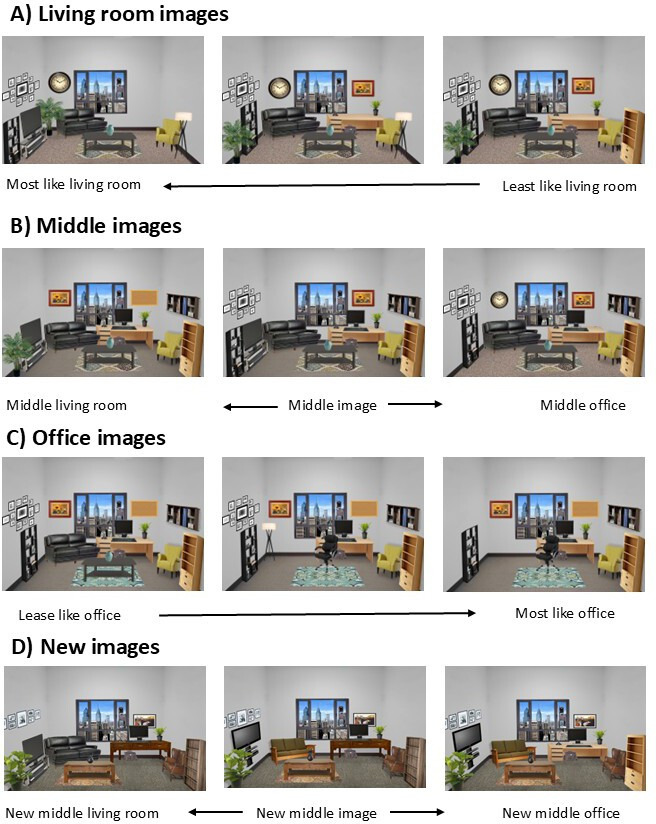
Continuum of contextual images presented during the Context Discrimination and Recognition Task (CDRT). (A) Three images contained most of the items from the original living room image and fewer items from the office image presented during the encoding phase. (B) Three images contained a comparable number of items from both the living room and office and were defined as indistinguishable images that were equally similar to both target rooms. (C) Three images contained most of the items from the original office image and fewer items from the living room image presented during the encoding phase. (D) Three images contained new versions of items from both the living room and office images that participants had not previously viewed; these images were defined as new images.

A continuum was created to depict a series of incremental images between the living room and office target images. By systematically manipulating the proportion of items from the living room and office in each image, we created a continuum consisting of 9 “signal strengths,” ranging from 0.1 (lowest office signal: 10% office items) to 0.9 (highest office signal: 90% office items; Figure 2A). Since we were specifically interested in assessing the discrimination of ambiguous images, we did not include the original target images (0% and 100% office items). At a signal strength of 0.5, the image contained equal proportions of living room and office items from the original target images, making this image the most ambiguous.

##### Foil Images

Three foils were created as distractors, depicting novel images containing 12 items prototypical of an office and a living room. One foil contained a majority (n=10, 83%) of never-seen living room items and a minority (n=2, 17%) of items from the living room target image, another contained equal proportions of never-seen office and living room items (100% never-seen items), and one contained a majority (n=10, 83%) of never-seen office items and a minority (n=2, 17%) of items from the office target image (Figure 2B). Each foil (living room, middle, and office) was presented an equal number of times.

### Task Design

Participants first completed an encoding phase (Figure 1A) that involved passively viewing each of the 2 target images (ie, office and living room images), 2 times each (presented one at a time in a randomized order) for 5 seconds each. During a test phase (Figure 1B), participants were then presented with the images from the living room-office continuum and foil images and were instructed to indicate whether each image was “most similar to the office” target image, “most similar to the living room” target image, “indistinguishable” (ie, contained equal elements of both target images), or a “new” image (ie, a different room from either target image). Four response options (office, living room, indistinguishable, and new) were presented at the bottom of the screen to correspond to these selections. The target images themselves were not presented during the test phase. Participants made their responses via button press. Interstimulus intervals ranged from 1 to 9 seconds (random, square distribution) and consisted of a white fixation cross on a black screen. Each of the 12 images (9 test images and 3 foils) was randomly presented 12 times over the course of 3 blocks (all images were presented 4 times per block), resulting in a total of 144 trials.

### Data Scoring

#### Discrete Scoring

##### Categorical Performance Metrics

We calculated discrimination and recognition scores consistent with prior methods examining discrimination and recognition of simple items [22]. The 9 continuum images (living room-office) were grouped into three categories: (1) “more like the living room” (the 3 images that contained the most items from the living room target [90/10, 80/20, and 70/30 living room item distributions]); (2) “middle” images (the 3 most ambiguous images with 40/60, 50/50, and 60/40 office vs living room item distributions); and (3) “more like the office” (the 3 images that contained the most items from the office target [90/10, 80/20, and 70/30 office item distributions]; Figure 2). The number of correct living room trials was defined as the sum of trials where one of the 3 images in the living room “grouping” was correctly categorized as “Living room.” The number of correct office trials was defined as the sum of trials where one of the 3 images in the office “grouping” was correctly categorized as “Office.” Because the proportion of items from each target image was equal or nearly equal for the middle images, the number of correct middle trials was defined as the sum of trials where one of the 3 middle images was correctly categorized as “indistinguishable.” The number of correct new trials was defined as the sum of trials where one of the 3 foil images was categorized as “New.”

##### Discrete Discrimination Score

Discrete Discrimination Score (DDS) was calculated to estimate PS processes, consistent with prior studies [22], by summing the correct new (foils labeled as “New”) trials and subtracting the number of errors—“living room” and “office” trials labeled as “New”—to correct for response bias. In order to yield a proportional value, this score was divided by the total number of new trials.


$$DDS=\frac{\text{number of correct new trials}}{\text{total number of new trials}}-\frac{\text{number of living room and office trials labeled “new”}}{\text{total number of living room and office trials}}$$

##### Discrete Recognition Score

Discrete Recognition Score (DRS) was calculated to estimate PC processes, consistent with prior studies [22], by summing correct living room and office trials and subtracting the number of errors—new trials labeled as “Office” or “Living room”—to correct for response bias. In order to yield a proportional value, this score was divided by the total number of living room and office trials.


$$DRS=\frac{\text{number of correct living room and office trials}}{\text{total number of living room and office trials}}-\frac{\text{number of new trials labeled “living room” or “office”}}{\text{total number of new trials}}$$

Including only living room and office images in these calculations yielded recognition scores that captured the perception of greatest similarity to one of the target images. These scores reflected the recognition of a specific scene (ie, the office or living room scene) rather than the recognition of discrete items that make up a scene. However, middle trials also required the recognition of items previously seen in each target image (ie, not “new” items) and, therefore, can be included in recognition performance. Therefore, a second version of this formula was computed with the ambiguous middle trials to yield a Discrete Recognition Score (Middle Trials; DRSmid).


$$\begin{array}{c} {DRS}_{mid}=\frac{\text{number of correct living room and office trials }+\text{ number of correct middle trials}}{\text{total number of living room and office trials }+\text{ total number of middle trials}}\\ -\frac{\text{number of new trials labeled “living room” or “office”}}{\text{total number of new trials}} \end{array}$$

### Psychophysical Scoring

#### Psychometric Modeling of Perceptual Bias and Sensitivity

In addition to traditional discrete discrimination and recognition scores, we aimed to capture more fine-tuned aspects of discrimination of complex scenes across the living room-office continuum using a psychophysics approach. A psychometric curve (logistic function) was fitted to the response data of each participant to derive 2 metrics shown to characterize unique aspects of perceptual processing in the context of ambiguity [26]: bias and sensitivity. *Bias* is derived from the threshold of the logistic curve (ie, *x* [office signal strength, ranging from 0.1 to 0.9] when *y*=50% “office” responses), and here, indexes the “tipping” point of the function where the perceptual system shifts from the meaningful recognition of one target to another. In the context of the CDRT, a typical threshold estimate less than 0.5 represents a bias toward the living room and a threshold more than 0.5 represents a bias toward the office. However, we were interested in bias toward either context without regard for the direction, so *absolute bias* was calculated by taking the absolute value of each participant’s threshold deviation from 0.5 (ie, |(Thres–0.5)|), with higher values indicating stronger bias toward either the office or living room. *Sensitivity* was determined using the width of the logistic function, calculated as the projection of *y*=5% to 95% onto the x axis. In the case of the CDRT, lower width estimates indicate greater sensitivity to changes along the continuum.

#### Response Coding

Response data for each participant were preprocessed using custom Microsoft VBA code, which summed (1) the total number of office responses per signal strength across the living room-office continuum and (2) the total number of trials per signal strength (maximum=12). A deterministic approach was used to recode each “indistinguishable” responses. For each participant, the first occurrence of an “indistinguishable” response was assigned to “Office,” the second occurrence to “Living room,” and the third occurrence to “Office,” and so on, and these were added to the total office endorsements for their corresponding signal strength. Trials in which the participant did not respond or responded “New” to a nonfoil test image were excluded.

#### Parameter Estimation

We used the psignifit 4 (Wichmann Lab, University of Tübingen) toolbox [27] implemented in MATLAB (R2019a; Mathworks) to estimate the 2 free parameters—*m* (threshold, ie, *x* value at *y*=0.5) and *w* (width of the function, ie, projection of *y*=0.05 to 0.95 onto *x*)—of the logistic function for each participant:

,$$S_{logistic}\left(x;m;w\right)=\;\frac{1}{1+e^{-2\log\left(\frac{1}{.05}\;-\;1\right)\;\frac{x-m}{w}}}$$

where *S*_logistic_ refers to the logistic sigmoid function, *x* is the stimulus level, *m* is the threshold, and *w* is the width of the function. By default, psignifit 4 also includes other parameters (eg, guess rate and lapse rate) to allow more flexible shapes of psychometric functions; here, we fixed them at 0. Parameters were estimated using Bayesian inference, such that prior distributions of the parameters are specified to constrain the estimates to reasonable values. We used the default priors for both *m* and *w*. Default prior for *m* is a uniform distribution over the range of the *x* values of the data (ie, 0.10-0.90) with a cosine falloff to 0 over half this data range (ie, at −0.3 and at 1.3). Default prior for *w* is a uniform distribution between 2 times the minimal distance of 2 tested stimulus levels (ie, 2×0.833%=0.167) and the range of the stimulus levels (ie, 0.90‐0.10=0.80), with cosine falloffs to 0 at 0.833 (the minimal difference of 2 stimulus levels) and at 2.40 (3 times the range of the tested stimulus levels). The visual inspection of individual curve fits suggested an overall good fit.

### Statistical Analysis

#### Power Analysis

An a priori power analysis was conducted using the software package G*Power (University of Kiel and Heinrich Heine University Düsseldorf). Given that no data had been previously collected on this task, we assumed a moderate effect size and determined that a total sample of 40 participants would provide 88% power to detect relationships between behavioral performance measures with a type 1 error rate of 5%. A total of 48 participants completed the task. Four participants were removed from all analyses due to excessive nonresponding (missed more than 25% of trials for one of the image categories; n=1) and random responding (n=3). Thus, our final sample of 44 participants provides adequate power to test questions of interest. In addition, all analyses include adequate information to determine observed power (either correlation coefficients or partial *η*^2^).

#### Data Analysis

Primary analyses were performed in MATLAB (R2019a) to quantify DDS, DRS, bias, and sensitivity. Pearson correlations and ANOVAs were conducted in SPSS (IBM version 30) to examine relationships between DDS, DRS, bias, and sensitivity on the CDRT. Given our goals to quantify typical performance on this task and test independent relationships between various aspects of discrimination and recognition of complex scenes, we did not correct for multiple comparisons.

## Results

Across our sample, DDS, DRS, DRSmid, bias, and width scores met the assumptions of normality.

Age was negatively associated with the DRS score (*r*=−0.34, *P*=.03) and the DRSmid score (*r*=−0.33, *P*=.03). Age was not associated with DDS, bias, or width (*P* values>.10), and scores did not differ across participants based on categories of gender (*P* values>.17), race/ethnicity (*P* values>.44), or education level (*P* values>.27). Given that the goal of this paper was not to examine relationships between performance and age, we controlled for age in all subsequent analyses to specifically focus on relationships between our measures of interest.

**Figure 3.**
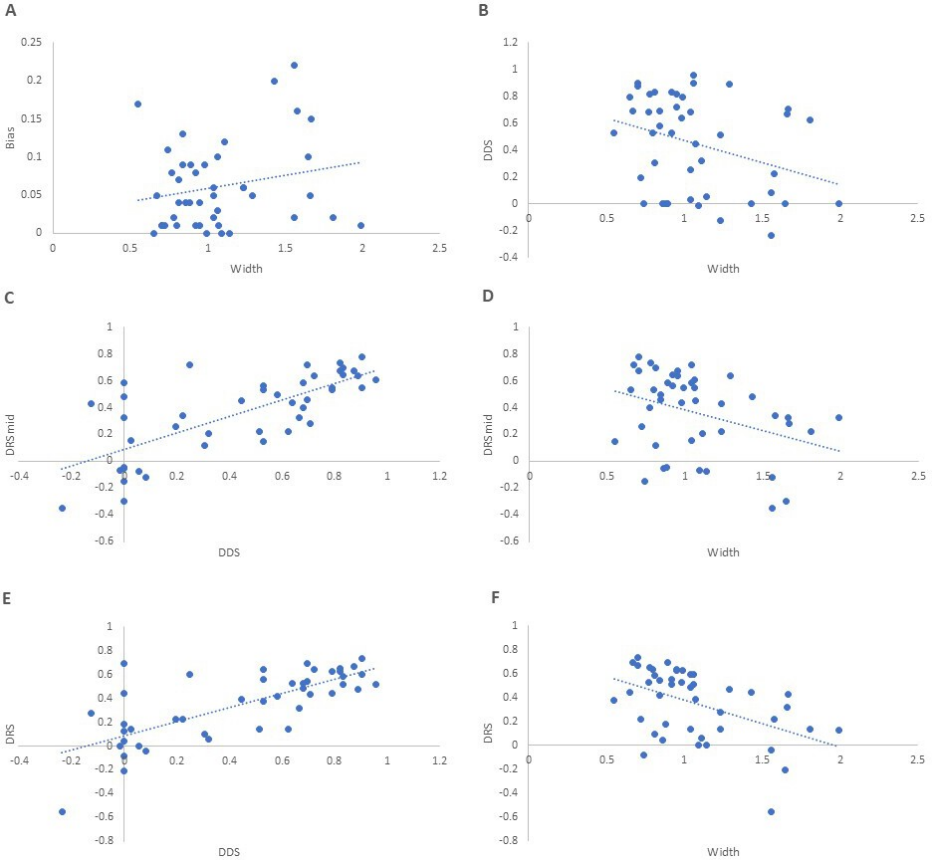
Relationships between scoring metrics. (A) Width vs bias, (B) width vs DDS, (C) DDS vs DRSmid, (D) width vs DRSmid, (E) DDS vs DRS, and (F) width vs DRS. DDS: Discrete Discrimination Score; DRS: Discrete Recognition Score; DRSmid: Discrete Recognition Score (Middle Trials).

For DDS and DRS, a score of 0 indicated a response bias–corrected accuracy of 0%, and a score of 1 indicated a response bias–corrected accuracy of 100%. Negative scores on these measures indicated a greater proportion of incorrect responses than correct responses (eg, more new images than living room/office images labeled as “living room,” “office,” or “indistinguishable”). DDS in our total sample ranged from −0.24 to 0.96 (mean 0.45, SD 0.36). DRS ranged from −0.56 to 0.74 (mean 0.35, SD 0.29). DRSmid ranged from −0.35 to 0.78 (mean 0.36, SD 0.30). A repeated-measures ANOVA examining the number of accurate responses across the 9 office signal strengths on the living room-office continuum revealed a significant gradient effect, *F*_8,344_=30.35, *P*<.001, *η^2^*=0.41, which was most prominently quadratic in nature, *F*_1,43_=31.29, *P*<.001, *η^2^*=0.42, suggesting that participants were more accurate at the ends of the continuum (ie, when images more closely resembled the target images). The results of a Pearson correlation controlling for age revealed a significant positive relationship between DDS and DRS scores, *r*(41)=0.71, *P*<.001, and between DDS and DRSmid, *r*(41)=0.71, *P*<.001 (Table 2, Figure 3).

**Table 2.** Descriptive statistics.

Variable	Mean (SD)	Skew	Kurtosis	1	2	3	4
Discrete Discrimination Score (DDS)	0.45 (0.36)	−0.31	−1.40				
Discrete Recognition Score (DRS)	0.35 (0.29)	−0.93	0.73	0.71^a^			
Discrete Recognition Score Mid (DRSmid)	0.36 (0.30)	−0.73	−0.40	0.71^a^	0.93^a^		
Width	1.04 (0.33)	1.03	0.19	−0.31^a^	−0.47^a^	−0.35^a^	
Bias (absolute value)	0.07 (0.06)	1.79	4.18	−0.17	−0.06	−0.17	0.20

a Significant at *P*<.05.

Width ranged from 0.55 to 1.99 (mean 1.07, SD 0.35), with a smaller score indicating greater sensitivity to discriminate across the living room-office continuum. Absolute bias scores ranged from 0.00 to 0.22 (mean 0.06, SD 0.06). A bias score of 0 indicated no bias toward either end of the image gradient, whereas greater bias scores indicated a tendency to perceive ambiguous images as being more like the extreme ends of the continuum (ie, more like the office or living room). When controlling for age, width and bias scores were not correlated with one another, *r*(41)=0.20, *P*=.21.

When controlling for age, width was significantly correlated with DDS, *r*(41)=−0.31, *P*=.04; DRS, *r*(41)=−0.47, *P*=.001; and DRSmid, *r*(41)=−0.35, *P*=.02 (Figure 3), suggesting that better discrimination and recognition were related to greater sensitivity (ie, smaller values on the width index) to small changes across the living room-office continuum. To verify that the relationship between width and DDS was specific to discrimination rather than general memory performance, this correlation was conducted again, controlling for DRS in addition to age. The relationship between width and DDS was no longer significant, *r*(40)=0.05, *P*=.78. To verify that the relationships between width and DRS or DRSmid were specific to recognition rather than general memory performance, these correlations were conducted again controlling for DDS in addition to age. The relationship between width and DRS remained significant, *r*(40)=−0.38, *P*=.01, but the relationship between width and DRSmid did not, *r*(40)=−0.19, *P*=.23. When controlling for age, DDS and DRS were not correlated with bias (*P* values>.28).

**Figure 4.**
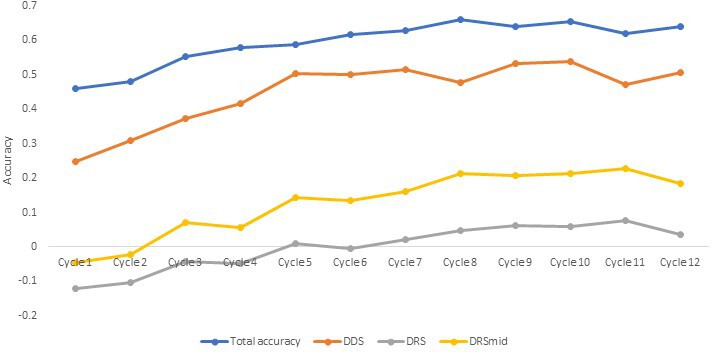
Accuracy across time. DDS: Discrete Discrimination Score; DRS: Discrete Recognition Score; DRSmid: Discrete Recognition Score (Middle Trials).

It is possible that learning and practice have an impact on task performance, such that encoding may continue throughout the multiple presentations of the images over the course of the task. If this is the case, it could make it difficult to isolate the processes of discrimination and recognition. To explore possible learning effects over the course of the task, we conducted a repeated-measures ANOVA with 12 levels representing the 12 cycles of the image presentations as the independent variable and the proportion of accurate trials as the dependent variable. Each of the 12 images was presented in random order in cycles, so all 12 images were presented once before repeating (eg, the first cycle contained the first presentation of each of the 12 images in random order, such as 3 “living room,” 3 “middle,” 3 “office,” and 3 “new,” followed by the second cycle with the same 12 images again presented in a new random order). Accuracy was defined as the proportion of images in each cycle that were responded to correctly (eg, living room as “living room,” office as “office,” middle as “indistinguishable,” and new as “new”). After controlling for age, the results revealed a significant effect of time, *F*_11,462_=2.57, *P*<.01, *η^2^*=0.06. The proportion of accurate trials ranged from 0.46 (cycle 1) to 0.66 (cycle 8) and 0.64 (cycle 12), suggesting an improvement in performance on the task over time (Figure 4). This pattern indicative of improved performance over time was replicated when we looked at DDS (*F*_11,462_=3.18, *P*<.01, *η^2^*=0.07) but not DRS (*F*_11,462_=0.86, *P*=.58, *η^2^*=0.02) or DRSmid (*F*_11,462_=0.82, *P*<.06, *η^2^*=0.02).

## Discussion

### Principal Findings

This study designed and tested a novel context processing task, the CDRT, establishing benchmarks for performance on indices of discrimination and recognition, reflecting the PC/PS balance in hippocampal processing of complex scenes. These results pave the way for studying established context-processing deficits and associated “neural signatures” of these deficits in psychiatric disorders [1-3,5,6,9,10,23]. While existing tasks have focused on discrimination and recognition of simple items, the CDRT addresses a gap in the literature and an urgent need to assess contextual processing by providing a tool and a benchmark for assessing these processes with more ecologically valid contextual stimuli. Moreover, while previous tasks are well-suited to quantify discrimination of simple items as a proxy for PS [22,28-30], our task is particularly well-suited to assess context-based PC, testing the recognition of complex scenes.

The results indicate that the CDRT is sufficiently sensitive to capture a range of performance scores across a continuum of contextual scenes. A key strength of this task is its ability to assess both PS- and PC-like processes within a single, ecologically valid paradigm. Increased accuracy on trials with a larger number of target items (ie, items that more closely resembled the target images), as compared to those with less shared items, supported the validity of this task in assessing context PC. The better ability to discriminate new from previously seen complex scenes (ie, DDS) was associated with better ability to correctly identify previously seen complex scenes based on partially ambiguous information (ie, DRS).

In addition to examining discrete discrimination and recognition scores, we also used a psychophysics function [26] to measure sensitivity to the image continuum (ie, width) and response bias toward either end of the continuum on the CDRT. Both discrete discrimination and recognition accuracy were associated with greater sensitivity to differences across a continuum of ambiguous complex scenes (ie, narrower width). While tasks such as the MST include a recognition index, this index best captures recognition memory for previously seen objects rather than the PC-like process of identifying a whole object based on partial information [15,22]. Because the CDRT uses a *gradient of previously seen items from complex scenes*, it is uniquely well positioned to probe nuances in PC-based performance.

### Limitations

This study has several limitations that should be considered when interpreting the findings. For one, response accuracy improved over time, indicating an effect of learning during the test phase. This learning effect suggests that participants may have continued encoding information about the target images during the test phase, in addition to utilizing their memory for what was presented in the encoding phase. Therefore, it is possible that our indices of recognition (ie, DRS) may have also captured the results of additional encoding processes. Future studies using this task could more effectively isolate discrimination and recognition by examining performance at only early or late intervals of the test phase. During task administration, we recommend an initial training phase to eliminate novelty effects or the addition of washout periods between task sessions to reduce the immediate, short-term impact of previous testing. Moreover, in statistical analyses, cycle numbers could be included as fixed effects in linear mixed models. Variations on this task could also be considered to avoid repeated presentations of the same stimuli (eg, more target images and fewer trials). Additionally, it is possible that, instead of PC, participants could be using gist memory to engage in recognition. Specifically, the target images were commonly recognizable scenes (ie, an office or living room); therefore, recognition performance on this task may have been influenced by getting the gist of the type of room based on elements contained in the target images (eg, participants identify that the image looks like a living room but not necessarily the specific living room they saw before [31]). However, this was addressed by subtracting trials when participants identified the “new” living room as “living room” in the DRS calculation, thus correcting for a general tendency to call any stimulus that looks like a “living room.” Furthermore, although stimuli were designed to be generic and nonemotional, these rooms might not be perceived the same way across all cultures, leading to individual differences in memory. Lastly, we did not assess the degree to which participants encoded target images holistically or as individual items from each image, and thus, we could not examine the potential effect that variability in encoding may have had on performance during the test phase.

### Future Directions

Despite these limitations, this study established performance on the CDRT in healthy participants and laid the groundwork for future studies to examine the utility of the CDRT in clinical samples and in interrogating associated neural circuits. While these findings provide preliminary evidence that these metrics effectively capture memory performance in healthy individuals, future research should further characterize these effects with larger samples to ensure the robustness of the findings. Given previous evidence of contextual memory deficits in PTSD [4,19,20] and differences in task performance between trauma-exposed and nonexposed participants [32], one possible application of this task could be to examine differences in contextual processing in individuals with PTSD compared to healthy individuals with and without trauma exposure. Future work could also use these scoring metrics to examine how the balance of PS and PC, and potential biases toward overcompletion or overseparation, may relate to clinical conditions such as PTSD. Furthermore, memory deficits associated with clinical conditions such as PTSD and depression have been linked to functional and structural abnormalities in the hippocampus [33-38]. Thus, future applications of this task should also examine context-based encoding and retrieval processes, and associated hippocampal function, to understand both adaptive and maladaptive contextual modulation processes. Crucially, this work should characterize how hippocampal interactions with broader neural networks support successful recognition and discrimination. Future studies examining task performance for clinical samples may also investigate possible differences associated with state-based affect or emotional valence. Additionally, given the relevance to fear and extinction learning in individuals with PTSD, a future direction involves modeling rates of learning over the task and examining these rates in relation to clinical symptoms. Future studies should also establish whether task performance on the CDRT is associated with neural functions in these regions and compare relationships between neural function and performance in clinical and nonclinical samples.

### Conclusions

The CDRT was developed to assess discrimination and recognition processes with respect to complex scenes. This novel task builds on existing tasks probing PS and PC for simple items. Our results in healthy adults demonstrate a range of performance scores and associations between discrete measures of discrimination and recognition with sensitivity to the continuum of complex images. Given the relevance of contextual information processing to a variety of psychiatric and neurodegenerative conditions, the CDRT offers a means for assessing deficits in contextual processing in healthy and clinical populations.

## Abbreviations

CDRT: Context Discrimination and Recognition Task
DDS: Discrete Discrimination Score
DRS: Discrete Recognition Score
DRSmid: Discrete Recognition Score (Middle Trials)
*DSM-5*: *Diagnostic and Statistical Manual of Mental Disorders, Fifth Edition*
MST: Mnemonic Similarity Task
PC: pattern completion
PS: pattern separation
PTSD: posttraumatic stress disorder
